# Encounter tool for Shared Decision Making about Adjuvant Treatment of Lung Cancer: Randomized Clinical Trial

**DOI:** 10.21203/rs.3.rs-8843093/v1

**Published:** 2026-05-11

**Authors:** Satya Sai Sri Bandi, Megan E Branda, Maria Mateo Chavez, Shubhangi Bagewadi, Aurora S. Norman, Victor Montori, Michael R. Gionfriddo, Iris G. Hargraves, Kasey Boehmer, Angela Stephen, Christi Sagen, Victor M. Montori, Juan P Brito, Konstantinos Leventakos

**Affiliations:** Mayo Clinic; Mayo Clinic; Mayo Clinic; Mayo Clinic; Mayo Clinic; Mayo Clinic; Geisinger College of Health Sciences; Mayo Clinic; Mayo Clinic; Mayo Clinic; Mayo Clinic; Mayo Clinic; Mayo Clinic; Mayo Clinic

## Abstract

**Background:**

Lung cancer is the leading cause of cancer-related death, and the most common cancer diagnosed globally. Approximately 85% of lung cancer cases are non-small cell lung cancer

(NSCLC). After initial surgical treatment, adjuvant therapy with conventional or targeted chemotherapy can help optimize survival. These treatments, however, must be tailored to each patients’ problematic situation, including cancer characteristics and goals of care.

Shared decision making (SDM) is a method of care by which patients and clinicians work together to form plans of care that advance the patient’s problematic human situation. This method often requires evaluating alternative options, “trying them on” for fit given their practical demands and favorable and unfavorable effects. Tools used within the encounter can facilitate and support SDM conversations, yet none exist for considering care post-surgical treatment of NSCLC.

Therefore, we aimed to evaluate the effectiveness of a novel encounter tool for SDM, NSCLC Choice, compared to usual care at improving SDM and reducing decisional conflict for patients with resected early-stage NSCLC considering adjuvant treatment.

**Methods:**

We will conduct a parallel, patient-level randomized trial comparing NSCLC Choice with usual care. Patients will be eligible if they are adults with stage >1B NSCLC eligible for postsurgical adjuvant chemotherapy. Allocation will be central and post-consent. Patients will be blinded to our trial hypothesis, instead of being invited to evaluate an educational intervention. Our primary outcome is SDM quality, which we will assess using participant reported measures (patient SDM-Q-9) obtained immediately post-encounter and an observer measure estimated from video recording of trial encounters in both arms (OPTION12). With resources to conduct a 100-patient trial, we will have >80% power to detect a 12-point medium difference between arms in patient-reported SDM-Q-9. We will also assess the secondary outcomes of knowledge about the disease and the features of treatment options using an ad hoc questionnaire, decisional conflict using the Decisional Conflict Scale, and patient and clinician satisfaction using the metrics used in prior SDM intervention trials. We will also determine encounter length using video recordings.

**Discussion:**

In testing NSCLC Choice, we seek to determine the extent to which this intervention can improve care for patients with NSCLC considering adjuvant therapy after surgery. In addition, we expect to determine whether this intervention is satisfactory to participants and feasible in practice. If successful, this study will result in an evidence-based tool for promoting patient-centered conversations around NSCLC and cancer care those fits. Trial Registration: NCT06122064 ClinicalTrials.gov

## Administrative information

**Table:** 

**Title {1}**	Shared Decision- Making Encounter tool for Adjuvant Treatment of Non-Small Cell Lung Cancer: Randomized Clinical Trial
**Trial registration {2a and 2b}**	ClinicalTrials.gov under Registration No: NCT06122064
**Protocol Version {3}**	Version number: 2.0 finalized on March 5, 2025
**Funding {4}**	Emerson Collective, LLC
**Author details {5a}**	Satya Sai Sri Bandi, Megan Brenda, Shubhangi Bagewadi, Maria Mateo Chavez, Aurora S. Norman, Victor Montori, Michael R. Gionfriddo, Iris Hargraves, Kasey Boehmer, Angela Stephen, Christi Sagen, Victor Montori, Juan Brito Campana, Konstantinos Leventakos,
**Name and contact information for the trial sponsor {5b}**	Mayo Clinic, Rochester, United States
**Role of sponsor {5c}**	This funding source had no influence on the design of the study and will not have any role during its execution, analyses or interpretation of the data, as well as in decisions to submit results.

## Introduction

### Background and rationale {6a}

Lung cancer is the leading cause of cancer-related deaths, and the most common cancer diagnosed globally (1). Approximately 85% of lung cancers are non-small cell lung cancer (NSCLC)(2). The treatment of NSCLC may include the use of surgery along with conventional or targeted chemotherapy. Current guidelines recommend that the specific treatment used should be tailored to patients’ cancer characteristics. However, patient-centered cancer care requires also tailoring based on what matters to the patient. Engaging patients in shared decision making (SDM) conversations to determine what matters and what to do about a problematic situation can improve various outcomes including knowledge, satisfaction, and decision quality (3). While many tools exist to support SDM conversations, none exist to facilitate conversations around post-surgical treatment for patients with NSCLC. Therefore, we aimed to evaluate the effectiveness of a novel encounter tool for SDM, NSCLC Choice, compared to usual care at improving decisional quality and reducing decisional conflict for patients with resected early-stage NSCLC considering adjuvant treatment. (4-11)

### Objectives {7}

#### Objective 1:

To estimate the extent to which NSCLC Choice improves SDM quality compared to usual care. **Hypothesis**: Using NSCLC Choice improves SDM quality.

#### Objective 2:

To estimate the extent to which NSCLC choice reduces decisional conflict compared to usual care. **Hypothesis:** Using NSCLC Choice reduces decisional conflict.

### Trial Design {8}

**Figure 1:**
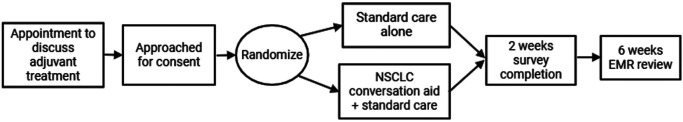
Study Flow: Eligible patients are consented and randomized to standard care alone or the NSCLC conversation aid plus standard care, followed by a 2-week survey and 6-week electronic medical record review.

We will conduct a patient-level randomized controlled trial. Participants will be randomly assigned in a 1:1 ratio to either NSCLC Choice or usual care. Figure 1 graphically illustrates the overall design of the study. All study procedures will be approved by an institutional review board (IRB # 23-003089). The trial is registered with ClinicalTrials.gov (registration number NCT06122064, registered on 30 October 2023). This protocol is reported following the SPIRIT (Standard Protocol Items: Recommendations for Interventional Trials) guidelines (see Additional file).

## Methods: participants, interventions, and outcomes

### Study Setting {9}

The trial will take place in a single Medical Oncology Department at one Academic Medical Center.

### Eligibility criteria {10}

Clinicians (physicians, nurse practitioners and physician assistants) caring for eligible patients will also be eligible for participation.

We will recruit adult patients diagnosed with NSCLC stage >1B, and deemed eligible for adjuvant treatment by their oncologist, with upcoming in-person or virtual appointments for cancer care with a participating clinician. We will exclude patients who have significant barriers to providing written informed consent or engagement in SDM, such as those patients with dementia or severe hearing or visual impairments.

#### Training personnel

After clinicians are consented, study personnel will demonstrate the intended use of NSCLC Choice. Study personnel may re-train clinicians as needed or in response to deviations in the quality of delivery observed on video recordings.

#### Who will gather informed consent {26a} and {32}

Clinicians will be recruited either during department meetings or immediately prior to an encounter with an otherwise eligible patient. Study staff will review the study with the clinician and if the clinician agrees to participate, obtain written informed consent. Study staff will approach eligible patients prior to their visit, assess their interest, and obtain written informed consent if the patient agrees to participate.

A copy of the consent form is attached as Additional file 4.

#### Consent or assent: ancillary studies {26b}

Participants may optionally consent to the future use and sharing of their data and audio/video recordings for ancillary research, as specified in the informed consent form found in additional files 4 and 5.

#### Interventions

### Explanation for choice of comparators {6b}

The comparator for this study is usual care, representing the current usual practice without the use of NSCLC Choice. This approach enables a direct assessment of the extent to which NSCLC Choice improves the quality of the decision-making process and reduces patient decisional conflict in comparison to usual clinical encounters.

#### Intervention description {11a}: NSCLC Choice Conversation Aid

**Figure 2:**
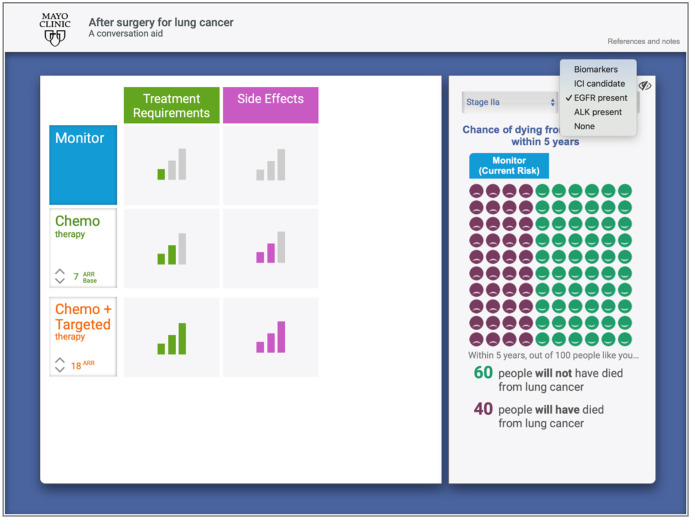
NSCLC Choice Aid

The NSCLC Choice tool was developed using a human-centered design approach refined by our group over 20 years(12). Initially, we observed 13 relevant encounters to identify the challenges patients and clinicians face in deciding on post-resection adjuvant therapy for NSCLC. Based on the observations we created an initial prototype that aimed to reduce those challenges and had clinical champions use it in real, relevant encounters. Based on those observations and participant feedback, the prototype was iteratively revised and field-tested until it was judged useful, usable, and desirable by clinicians, patients, and the study team. The finalized tool (Figure 2) includes a calculator of the patient’s risk of dying within 5 years depicted in a 100-person pictograph. This shows an approximate proportion of patients who will and will not die within 5 years if they, subject to relevant clinical indications, receive (1) chemotherapy, (2) chemotherapy plus immunotherapy, (3) targeted therapy, or (4) monitoring for disease progression alone. The tool, the options it presents, and its risk display can be individualized to the patient based on PDL1>1%, EGFR mutation, and the TNM system staging. It also supports the discussion of practical considerations and adverse effects of the various options. The tool is regularly revised based on updates of clinical practice recommendations and accruing outcomes data from ongoing clinical trials. For example, as of March 2025 it includes information for use of observation, chemotherapy, immunotherapy, EGFR inhibitor and has been updated to include an adjuvant ALK inhibitor which recently received FDA approval.

The most up-to-date version of the encounter tool is available After surgery for lung cancer

### Mode of use {11d}

NSCLC Choice is designed as an encounter-based conversation aid. It was designed to support conversations rather than enforce scripted discussions. As such, the tool allows for flexibility in how and when the tool is used during the visit. Clinicians may also choose not to use the tools with certain enrolled patients, based on their professional judgment. As such we plan to assess the fidelity of use which captures not only if the tool was used but also if it was used as intended to support conversation. An assessment of intended use will be based on a previously developed instrument which will be adapted to NSCLC.

#### Usual care encounters

In usual care encounters, the clinician will conduct the encounter according to their standard care practices. The clinician will be notified prior to entering the room the patient’s allocation and if the patient is allocated to usual care will be told not to use the tool. To monitor contamination (i.e., use of the tool in patients allocated to usual care) we will video record the visit and analyze whether the tool was used as well as the extent to which behaviors supported by the tool were present. To conduct this analysis, we will modify the Observed SDM Implementation Measure (OSIM) to be specific to NSCLC Choice(13).

### Criteria for discontinuing or modifying allocated interventions {11b}

Participants may discontinue or modify their participation if they experience major barriers to continuing (e.g., progression of disease, inability to complete follow-up).

### Strategies to improve adherence to interventions {11c}

To maximize clinician fidelity to the intended use of the tool we will train each participating clinician, periodically check video recordings for fidelity, and provide feedback and retraining as necessary.

### Interventions: concomitant care {11d}

All other care and interventions, as determined by the treating clinician, will be permitted during the trial.

### Provisions for post-trial care {30}

No provisions for ancillary or post-trial care will be provided, as the study does not involve any physical interventions.

### Outcomes {12}

Patient outcomes, Primary Endpoints:

To assess the primary endpoint of SDM process quality, two measures will be collected.

#### SDMQ-9:

a self-reported 9 item questionnaire that assesses the effectiveness of the intervention on promoting SDM. Each item has a six-point Likert-type scale that ranges from 0 (Completely disagree) to 5 (Completely agree). The overall score is the sum of the 9 items, where lower values indicate less perceived SDM, and higher scores indicate more perceived SDM (14).

#### OPTION12:

an observer scale applied to recorded encounters which assesses the degree of effort the clinician made to involve patients in SDM. The scale consists of 12 items scored from 0, no effort to 4, exemplary effort. The 12 items are summed and converted to a 100-point scale. Initially, a sample of 20% of the recordings will be assessed by two or more reviewers. Agreement will be assessed by Lin’s concordance index, where any value over 0.8 will be considered concordant. Once the agreement is reached, other videos will be reviewed. If concordance does not occur within the first 20% encounters, the two reviewers will assess cases of difference and review an additional 10 cases to test for agreement. Recordings scored by both reviewers will be averaged (15).

#### Patient Outcomes:

Secondary endpoints

#### Knowledge transfer:

estimated by asking 6 investigator-developed questions to patients about treatment options to prevent recurrence of lung cancer. The 6 questions use a response format of “True / False / Do not know.” Correct responses will be summed up and divided by the total number of questions asked. If a patient answers at least 1 knowledge question then they will be assessed for this outcome, where all missing responses will be coded as incorrect.

#### Patient Satisfaction with clinical encounter:

assessed with 1 question on a 7-point Likert scale. Patients will be asked whether they would recommend the approach used to others facing similar discussions.

#### Decisional Conflict Scale (DCS):

A 16-item scale assessing respondent uncertainty about a course of action. Each of the 16 DCS items is scored on a 0-4 Likert-type scale; the items are summed, divided by 16 and then multiplied by 25. The scale is from 0-100 where higher scores are reflective of uncertainty about the choice. There are 5 DCS subscales, where a DCS subscale consists of 3 questions (1 subscale of 4). If 2 of 3 (or 3 of the 4) questions within a subscale have responses, then the patient would be considered as a responder, and a score could be calculated. If more than one response per subscale is missing, then that specific subscale is not calculated for the patient. An overall DCS score can be calculated if no more than 5 responses are missing if each missing response falls into a different subscale. The entire scale will be administered 2 weeks post-encounter of interest and the subscales of informed, and uncertainty will be administered post encounter (16).

#### Decision made on treatment:

derived from the recorded encounter. Decisions about whether and which therapy to follow may not be made within the encounter as the patient may wish to take some time to decide or may be contingent on test results not yet in hand (e.g. tumor mutation). In these cases, or if the final decision is not clear in the video recording, we will extract it from clinical documentation in the electronic health record (EHR).

### Clinician Outcomes

#### Satisfaction with the encounter:

assessed with 2 questions immediately after the patient encounters. A 5-point Likert-type scale will assess satisfaction with discussion about NSCLC choice. The clinician will also be asked whether they would recommend the approach used to other clinicians using a 7-point Likert-type scale (17).

### Participant timeline {13}

Patient’s participation in the study involves completing two questionnaires by each participant: one at baseline after the encounter of interest is complete, one at 2 weeks post enrollment, and EHR is reviewed at 6 weeks post enrollment (Figure 1). The initial questionnaires will either be provided in person or sent via email, based on the participant’s preference. Subsequent surveys will be automatically sent to the participant's email. The estimated time to complete each questionnaire is approximately 10 minutes.

### Sample size estimation {14}

The targeted sample size is 100 patients. SDMQ-9 has an estimated mean value of 70 at baseline, with a standard deviation of 20. Assuming an average cluster size of 5 patients per clinician and 20 clinicians, along with an intra cluster correlation coefficient (ICC) of 0.01, we would achieve 82% power to detect a 12-point increase in the intervention group (N=50 per group), using a two-sided test with an alpha of 0.05. If 10 clinicians are recruited, with an average cluster size of 10 patients per clinician, we would have 80% power.

### Recruitment {15}

#### Clinician Enrollment

The principal investigator will present the study overview in either department meetings or in one-on-one conversations with eligible clinicians. If the principal investigator is not available, then the study coordinator or another member of the study team will reach out to the clinician to see if they are interested in participating. If the clinician is interested, then a member of the study team will review and have them complete the consent.

#### Patient Enrollment

The study coordinator will scan upcoming appointments for enrolled clinicians in the EHR for potential eligible patients. After screening, if a patient appears to be eligible, they will contact the clinician to confirm. Once confirmed the study coordinator may reach out to the patient by one of the following methods: 1) Meet with patients as they are roomed to discuss the study and review the consent process, 2) Contact the patient by phone to see if they would be interested in participating, or 3) Contact the patient through the patient portal.

The study coordinator will consent both clinician and patient for video/or audio recording of the encounters. Any adult who accompanies the patient into the exam room for the appointment will be orally consented to being video/or audio recorded.

Patient Consent: Patients’ consent may be completed in person or online depending on the means of recruitment. A copy of the consent form is attached as Additional file 5.

### Allocation: sequence generation {16a}

#### Patient allocation:

Eligible patients will be allocated at 1:1 ratio into either usual care alone or NSCLC Choice. Block randomization will be completed using random block sizes stratified by clinician. The randomization will be carried out by a statistician using the REDCap platform (18, 19)

### Allocation Concealment mechanism {16b}

The allocation sequence will be concealed using the REDCap platform (18, 19) and is not accessible to be viewed or modified.

### Allocation Implementation {16c}

Initially, patients will be enrolled by study coordinators who are unaware of the study arm to which the patient will be assigned. Once a patient is enrolled and has provided written or electronic consent, the study coordinator will record the data in REDCap. Following this, participants will be randomized into one of the two arms of the study based on the randomization sequence.

### Assignment of interventions: Blinding

#### Who will be blinded {17a}

The clinician will not be blinded to the arm assignment due to the nature of the intervention. The patient is made aware of an educational intervention that may be used in the encounter, staying blind to the hypotheses of the trial. Study personnel collecting outcome data from the recordings and the study analyst will not be blind to the trial arm.

#### Procedure for Unblinding if needed {17b}

This study is not blinded; therefore, no unblinding procedures are applicable.

### Data Collection and Management

#### Plans for assessment and collection of outcomes {18a}:

Patients who are approached by study staff and agree to participate will be recorded in REDCap.

Potential patients who are found to be ineligible or those who decline participation will be documented in a recruitment tracking log, including reasons for ineligibility or decline, along with the patient’s age, sex, and race/ethnicity.

Self-reported data from patients and clinicians will be collected at multiple timepoints throughout the study (Table 1). At enrollment, clinicians will complete a demographic survey that includes questions on age, gender, race, ethnicity, and years in practice. After the index encounter, both patients and clinicians will complete a survey at the clinic, facilitated by the study coordinator or site-appointed staff. For virtual encounters, the survey will be provided electronically for completion. The post-encounter survey for patients will collect patient demographics, SDMQ9, knowledge, decision made, satisfaction, DCS, health literacy(20), and numeracy (21). If the encounter is in-person and the patient requests a return envelope, one will be provided to mail the completed survey back. If the survey is not returned within 10 days, a reminder will be sent with a new copy of the survey and a return envelope. A courtesy reminder call will be made within 5 days after the mailing. For virtual encounters, the study coordinator will join the session at the end to present the survey and assist the patient in completing the online form. If the patient cannot stay on the call, a paper survey will be mailed following the same procedure as for in-person encounters. Every effort will be made to complete the survey immediately after the encounter in the clinic for the most accurate data collection.

Patients will also receive a follow-up survey to be completed 2 weeks after enrollment, with the mode of completion based on patient preference (e.g., mail, online via email, or a portal message). Incomplete surveys will be conducted according to the same follow-up process described above.

Data from the medical records of all enrolled patients will be abstracted to capture demographic and clinical information, covering the period from before enrollment to 6 weeks after enrollment. Data collected on patients will include variables such as Eastern Cooperative Oncology- performance status(ECOG-PS), previous treatments for NSCLC, mutations, smoking history, marital status, education, race/ethnicity, and annual income (20, 21).

In addition to data captured through surveys and EHR data, we will also capture data through video or audio recording of the clinical encounter. For in-person encounters, the study coordinator will set up the portable digital video camera at the start of a visit where both the patient and clinician have consented. The camera will be aimed at the clinician’s desk, away from the physical examination table. The clinician and the patient will be instructed on how to occlude the lens, direct the camera to a wall or turn off the camera at any time they feel this is appropriate. After the visit the study coordinator will retrieve the recording device. For virtual visits the study coordinator will join the virtual visit to record the meeting and administer the survey. After the encounter, study coordinators will transfer the video file to a secure server for analysis, deleting the recording from the device after server confirmed backup. These recordings will be used to assess SDM (OPTION) as well as contamination and fidelity intended use (OSIM).

### Plans to promote participant retention and complete follow-up {18b}

Participants have the right to withdraw from the study at any time, for any reason. An expected dropout rate of approximately is <1% anticipated based on previous studies. To minimize dropouts, patients will receive phone or electronic reminders to complete the questionnaires.

### Data management {19}

The study will use the most current versions of all documents and forms, and the study team will follow the established study schedule. Data will be entered into the REDCap system, a HIPAA-compliant, secure platform that ensures validated data entry, edit checks, and logs of all data modifications. The statistical team will have access to the data at any time and can download it into statistical software for analysis. Data will be reviewed monthly by the statistical team to verify its accuracy and completeness. All data, documents, and analysis results will be stored within the health system, which is password-protected and backed up nightly.

### Confidentiality {27}

In this study, participant privacy will be safeguarded by ensuring that no names are used on any research data (including field notes, transcriptions of conference calls, meeting recordings, audio and video recordings of interviews, and transcripts). Participants will be assigned a unique study code number for identification. The link between the code number and participant identity will be stored in a secure, firewalled spreadsheet on a research server, accessible only to study investigators and study staff, all of whom have human subjects research certification. This spreadsheet will be deleted once all participant follow-up is complete. A do-not-call list will be maintained for individuals who decline participation to prevent multiple contact attempts, as patients may have several appointments at participating clinics during the study. All research materials will be stored securely on a server or in locked file cabinets, and all materials will be destroyed seven years after the study’s completion.

Digital recordings of clinical encounters will be immediately uploaded to the research team’s secure server and deleted from portable devices after overnight back-up of servers. The video and audio files will be identified using a code number that does not include the name of the clinician, support staff, or patient or reference to their medical record number or date of birth. The research data is only accessible with password protected and logged access.

### Plans for collection, laboratory evaluation and storage of biological specimens for genetic or molecular analysis in this trial/future use {33}

Not applicable, as no biological specimens are collected in this study.

## Statistical Methods

### Statistical methods for primary and secondary outcomes {20a} and {20c}

The study will be analyzed according to the *intention to treat principle (ITT)*, including all patients enrolled in the study and analyzed in the arm to which they were assigned, regardless of which intervention they received. Descriptive statistics will be conducted with means and standard deviations for continuous variables and counts and frequencies for categorical variables. We will use cluster (cluster at clinician level) adjusted t-test and chi-square test for comparisons between interventions and hierarchical generalized linear models (HGLMs) with random main effects specified at the clinician level when adjusting by more than arm. If clustering is not present, then the results will reduce to a model that assumes independence and reflects findings appropriately. Missing data will not be imputed for the analysis. Rates of missing data will be reported in the findings.

### Statistics: additional analyses {20b}

No planned additional analysis.

### Plans to give full access to the full protocol, participant level data and statistical code {31c}

The full study protocol will be available and published. Participant-level data will not be accessible due to privacy and confidentiality concerns, and a statistical analysis code will not be provided.

### Oversight and monitoring

#### Composition of the coordinating center and trial steering committee {5d}

This is a single center study conducted and managed by the oncology department within an academic medical center. The responsibilities within the research team are as follows:

Principal investigator/ Lead investigator: Responsible for overseeing study design, preparation of protocol including revisions, and identification and recruitment of clinicians.

##### Study coordinator:

Handles participant consent, data collection, clinical trials registration and communication with the IRB.

##### Statistician:

Responsible for data analysis.

The study team will meet weekly. No trial steering committee or stakeholder/public involvement group (nor any other committee) will be involved.

### Composition of the data monitoring committee, its role and reporting structure {21a}

No Data Monitoring Committee is required since the study does not include any medication, medical treatments, or procedures.

### Data monitoring: interim analysis {21b}

No interim analysis is planned.

### Adverse event reporting and harms {22}

Participating in the study does not pose any direct health risks and any potential risks to patients are expected to be minimal as the intervention is a conversation tool designed for use during clinic visits to assist patients in making decisions about adjuvant treatment. It does not offer recommendations without the clinician’s involvement and should only be used during a clinical visit where the clinician can provide context.

### Frequency and plans for auditing trial conduct {23}

Not applicable, as no independent auditing of trial conduct is planned.

### Plans for communicating important protocol amendments to relevant parties {25}

Any changes to the study plan which could impact how the study is carried out will require a formal update to the protocol. All members of the research team must agree on these changes, and they must be approved by the IRB.

### Dissemination Plans {31a}

The results of the study will be disseminated through peer-reviewed journals and presented at conferences.

## Discussion

This randomized clinical trial evaluates the efficacy of an SDM encounter tool in guiding adjuvant therapy decisions for patients with resected NSCLC. Adjuvant treatment decisions in lung cancer are complex, involving a balance of benefits, harms and practical considerations that affect both survival and quality of life (22). The best treatments are those that fit each person, advancing their problematic situation in ways that make sense to them(23). This trial could contribute to the field by estimating the extent to which an SDM encounter tool can enhance usual care of patients with early-stage NSCLC.

## Limitations

The field of adjuvant treatment for resected lung cancer is constantly evolving. We expect the evidence to evolve throughout this study. To ensure the protocol remains current, the team will convene quarterly to update the tools in line with the latest evidence and FDA approvals. If issues arise with participant accrual, we plan to extend the study to additional academic centers. This study is small, and estimates may prove insufficiently precise, and subgroup analyses, which have not been planned, may not be feasible. And yet, this trial, if successful, will provide important evidence to support the conduct of a larger multicenter trial using similar methods as those described here.

## Supplementary Material

This is a list of supplementary files associated with this preprint. Click to download.

AdditionalFile2.pdf

AdditionalFile3.pdf

AdditionalFile1.docx

AdditionalFile4.docx

AdditionalFile5.docx

## Figures and Tables

**Table 1:** Calendar of Events

	Prior to Study Enrollment	Appointment with Physician (Index Encounter)	Post appointment	2 weeks post- appointment	6 weeks post- appointment
**Patient Forms/Schedule**					
Informed Consent	X				
Index Encounter Survey^1^			X		
Follow-up survey^2^				X	
EMR Review			X		X
Video recording^3^		X			
**Physician Forms/Schedule**					
Informed Consent	X				
Survey-Demographic	X				
Follow-up Survey^4^			X		

1 Patient demographics, SDMQ9, knowledge, decision made, satisfaction, DCS Informed subscale, DCS Uncertainty subscale.

2 Confirmation of decision made, DCS, satisfaction.

3 OPTION12

4 Satisfaction

## Data Availability

The final trial dataset will be accessible to the study investigators. There are no contractual agreements that limit access to the data.
